# Curcumin and Graphene Oxide Incorporated into Alginate Hydrogels as Versatile Devices for the Local Treatment of Squamous Cell Carcinoma

**DOI:** 10.3390/ma15051648

**Published:** 2022-02-22

**Authors:** Lorenzo Francesco Madeo, Patrizia Sarogni, Giuseppe Cirillo, Orazio Vittorio, Valerio Voliani, Manuela Curcio, Tyler Shai-Hee, Bernd Büchner, Michael Mertig, Silke Hampel

**Affiliations:** 1 Leibniz Institute of Solid State and Material Research Dresden, 01069 Dresden, Germany; b.buechner@ifw-dresden.de (B.B.); s.hampel@ifw-dresden.de (S.H.); 2 Center for Nanotechnology Innovation, Istituto Italiano di Tecnologia, Piazza San Silvestro 12, 56127 Pisa, Italy; patrizia.sarogni@iit.it (P.S.); valerio.voliani@iit.it (V.V.); 3 Department of Pharmacy Health and Nutritional Science, University of Calabria, 87036 Rende, Italy; manuela.curcio@unical.it; 4 Children’s Cancer Institute, Lowy Cancer Research Centre, University of New South Wales, High Street, Randwick, NSW 2052, Australia; ovittorio@ccia.org.au (O.V.); tshai-hee@ccia.org.au (T.S.-H.); 5 School of Women’s and Children’s Health, University of New South Wales, Kensington, NSW 2052, Australia; 6 ARC Centre of Excellence in Convergent Bio-Nano Science and Technology, Australian Centre for NanoMedicine, University of New South Wales, Kensington, NSW 2052, Australia; 7 Institute of Solid State and Materials Physics, Technische Universität Dresden, 01062 Dresden, Germany; 8 Institute of Physical Chemistry, Technische Universität Dresden, 01062 Dresden, Germany; michael.mertig@tu-dresden.de; 9 Kurt-Schwabe-Institut für Mess- und Sensortechnik Meinsberg e.V., 04736 Waldheim, Germany

**Keywords:** curcumin, graphene oxide, alginate hydrogels, hybrid hydrogels, ionic crosslinking, squamous cell carcinoma

## Abstract

With the aim of preparing hybrid hydrogels suitable for use as patches for the local treatment of squamous cell carcinoma (SCC)-affected areas, curcumin (CUR) was loaded onto graphene oxide (GO) nanosheets, which were then blended into an alginate hydrogel that was crosslinked by means of calcium ions. The homogeneous incorporation of GO within the polymer network, which was confirmed through morphological investigations, improved the stability of the hybrid system compared to blank hydrogels. The weight loss in the 100–170 °C temperature range was reduced from 30% to 20%, and the degradation of alginate chains shifted to higher temperatures. Moreover, GO enhanced the stability in water media by counteracting the de-crosslinking process of the polymer network. Cell viability assays showed that the loading of CUR (2.5% and 5% by weight) was able to reduce the intrinsic toxicity of GO towards healthy cells, while higher amounts were ineffective due to the antioxidant/prooxidant paradox. Interestingly, the CUR-loaded systems were found to possess a strong cytotoxic effect in SCC cancer cells, and the sustained CUR release (~50% after 96 h) allowed long-term anticancer efficiency to be hypothesized.

## 1. Introduction

The interest in graphene and the materials derived from it for biomedical applications has grown tremendously in recent years [1,2,3,4]. In particular, graphene oxide (GO) has been largely investigated as a promising therapeutic delivery vehicle because of its particular properties [5,6]. Aside from the high surface-area-to-volume ratio and the sp^2^ carbon layer that allows π–π interactions with hydrophobic molecules [7,8], its oxygen-rich functionalities can form hydrogen bonds and electrostatic interactions with hydrophilic molecules [9,10], providing new opportunities to effectively bind to almost any type of organic molecule [11,12]. Such functional flexibility makes GO a useful platform that is capable of controlling the release of a wide range of therapeutic molecules, including chemotherapeutics, peptides, genes, and naturally occurring compounds [13,14,15,16,17].

Among the plethora of naturally occurring molecules used in biomedicine, our interest was drawn to curcumin (CUR), a diferuloylmethane constituent of the yellow pigments isolated from *Curcuma longa*, which is well known for its antioxidant, radical-scavenging, antimicrobial, and anti-inflammatory properties [18,19]. It has also shown potent anti-proliferative effects against several cancers due to its ability to interfere with different cellular pathways involved in carcinogenesis [20,21]. Therefore, CUR has been tested both in vitro and in vivo against a large number of tumors [22,23,24], thus sparking its interest as a potential skin cancer treatment [25,26], whereby several studies have supported CUR as a potential candidate for the treatment of squamous cell carcinoma (SCC) [27,28,29]. 

SCC is an invasive and lethal epithelial malignancy representing approximately 20% to 25% of non-melanoma skin cancers [30]. It arises from the prickle–squamous cell layers of the epidermis, shows keratinocytic differentiation [31], and is capable of metastasis to regional lymph nodes. 

Although local excision of smaller cutaneous SCC is frequently performed with acceptable outcomes, the treatment of patients with multiple lesions or condemned skin, particularly in the head and neck, is a more difficult challenge [30,32,33]. Therefore, the use of bioactive agents to treat such conditions might be a promising solution through both systemic and local administration [34,35,36].

Although an enormous number of studies have enlightened readers about the outcomes of CUR on a molecular level, its use as an effective pharmaceutical lead has been challenging, with no satisfying results up until now due to its poor solubility, low bioavailability, and uncontrolled reactivity in vivo [37,38]. For these reasons, a proper topical administration of CUR on a skin area affected by SCC might be desirable. 

Providing an appropriate vehicle system for the controlled release of CUR in such applications is crucial, since high topical dosages may result in toxicity concerns [39]. Among the different delivery systems exploited in the literature, the peculiar features of GO can lead to the development of highly effective dosage forms with prolonged release [40]. 

In order to achieve an acceptable degree of stability and hydrophilicity, hybrid GO–hydrogel systems were proposed, which allow the combination of the favorable properties of both counterparts: the high hydrophilicity and biocompatibility of hydrogel systems and the high drug affinity of GO [41,42]. Among the polymeric materials adopted to enhance the stability of graphene-based carriers, alginate showed several interesting features [43].

Alginate is a natural polysaccharide found in brown algae; it is biocompatible, non-toxic, non-thrombogenic, and non-immunogenic, and it is approved by the Food and Drug Administration (FDA) for various medical applications [44,45]. Its capacity to form a stable and swelling hydrogel in the presence of divalent cations (e.g., calcium ions) through ionotropic gelation has made this polymer of great interest for multiple medical applications, including the fabrication of scaffolds for tissue regeneration, wound healing, and protein/drug delivery systems [46,47]. 

In this study, CUR-loaded GO sheets were blended into alginate polymer and crosslinked by means of calcium ions, resulting in a flexible and mechanically stable hybrid hydrogel film. Such drug delivery systems have been proposed as patches for external use and are capable of performing an effective and sustained release of CUR onto SCC-affected areas.

## 2. Materials and Methods

### 2.1. Synthesis of Hybrid Hydrogels

Graphene oxide (GO) powder was added to a 2.0% (*w*/*v*) sodium alginate (ALG) solution in double-distilled water (ddH_2_O) with a GO-to-ALG ratio of 2.0% by weight. The mixture was thoroughly stirred for 15 min and then sonicated using a horn-tipped ultrasonic probe with 20% amplitude (BANDELIN electronic GmbH, Berlin, Germany) for 15 min in order to crack the GO aggregates and obtain a fine dispersion. Thereafter, a selected amount of a 0.5% (*w*/*v*) solution of curcumin (CUR) in ethanol was added to obtain CUR-to-ALG ratios of 2.5, 5.0, and 7.5% (*w*/*w*) across separate experiments.

To obtain a platform for the physical crosslinking of the hybrid dispersion, a solution consisting of 1.0% *w*/*v* agar and 0.2 M calcium chloride (0.2 M) in ddH_2_O was heated up until boiling and left to cool in a Petri dish. The solution containing ALG, GO, and CUR was then gently poured and left to crosslink at 40 °C for 2 h. The obtained CUR@H_ALG-GO_ hydrogel was peeled off the agar gel below and washed in ddH_2_O to remove unreacted species. Afterwards, the hydrogel was dried at 40 °C for 12 h. The same approach without the insertion of CUR (H_ALG-GO_ and H_ALG_) or GO (H_ALG_ and CUR@H_ALG_) in the reaction feed was used to prepare the control samples.

All chemicals were purchased from Merck/Sigma Aldrich, Darmstadt, Germany.

### 2.2. Instruments

Attenuated total reflectance Fourier transform infrared spectroscopy (ATR-FTIR) spectra were recorded on an IFS/66v/S (BRUKER Optic GmbH, Karlsruhe, Germany).

Dynamic light scattering (DLS) analyses were performed with a 90 Plus Particle Size Analyzer (Brookhaven Instruments Corp, Holtsville, NY, USA) at 25.0 ± 0.1 °C with a laser operating at 658 nm while measuring the autocorrelation function at 90°.

Thermogravimetric analysis (TGA) was performed on an SDT Q600 (TA Instruments, Hüllhorst, Germany) under a nitrogen atmosphere with the following conditions: 10 mg initial sample weight, 10 mL min^−1^ N_2_ flow, 25–800 °C temperature range, and 10 °C min^−1^ heating rate.

Optical microscope images were taken with an Axio Imager.A2m (Zeiss, Oberkochen Germany).

Scanning electron microscope (SEM) images of samples were taken with a NOVA NanoSEM 200 [0–30 kV] (Thermo Fisher Scientific, Hillsboro, OR, USA) by depositing samples onto self-adhesive conductive carbon tape (Plano GmbH, Wetzlar, Germany).

Atomic force microscopy (AFM) images were taken with a Dimension Icon (Bruker) operating in the tapping mode, and they were examined with the Nanoscope Analysis software, version 1.8.

### 2.3. Evaluation of Water Affinity

The water affinity of the blank and hybrid hydrogels was evaluated by immersing weighted specimens of each sample (~5.0 cm^2^) in the swelling medium, which consisted of phosphate-buffered saline (PBS) solution (10^−4^ M, pH 7.4), at 37 °C. At predetermined time intervals, surface moisture was removed by blotting samples with a tissue, and the samples were weighed. The water affinity was expressed as the water content percentage (*WR*) according to Equation (1):

(1) $$WR=\frac{W_{s}-W_{d}}{W_{d}}\times 100$$

Here, *W_d_* and *W_s_* represent the weights of samples in the dried and swollen states, respectively.

All chemicals were purchased from Merck/Sigma Aldrich, Darmstadt, Germany.

### 2.4. CUR Release Profiles

In vitro CUR release profiles were determined with the dissolution method. In separate experiments, weighted specimens of ~1 cm^2^ of loaded hydrogels were immersed in 10 mL of releasing medium consisting of a PBS (0.001 M, pH 7.4)/ethanol mixture (6/4, *v*/*v*) and maintained at 37.0 ± 0.1 °C in a water bath with alternate shaking. At predetermined time intervals, 1.0 mL of release medium was withdrawn, filtered with Iso-DiscTM Filters PTFE 25–4 (25 mm × 0.45 μm, Merck/Sigma Aldrich, Darmstadt, Germany), and, to ensure sink conditions throughout the experiment, replaced with fresh release medium. The amount of CUR in the solution was determined by UV–Vis analysis on an Evolution 201 spectrophotometer (ThermoFisher Scientific, Hillsboro, OR, USA) operating with 1.0 cm quartz cells set at 430 nm using the calibration curve of CUR. The amount of CUR released was expressed according to Equation (2):

(2) $$\mathrm{CUR}\;release=\frac{M_{t}}{M_{0}}$$

where *M_t_* and *M*_0_ are the CUR amounts (mg) detected at time *t* and loaded into the hydrogels, respectively.

All chemicals were purchased from Merck/Sigma Aldrich, Darmstadt, Germany.

### 2.5. Cell Culture

Human bronchial epithelial cells (HBEpc) and human squamous cell carcinoma SCC-25 (HPV-negative) were purchased from the American Type Culture Collection (ATCC, Manassas, VA, USA). HBEpc were cultured in ready-to-use bronchia/trachea epithelial cell growth medium (Cell Applications) supplemented with 10% fetal bovine serum (FBS), while the SCC-25 cells were grown in a complete medium composed of a 1:1 mixture of Dulbecco’s modified Eagle’s medium and Ham’s F12 (Invitrogen) supplemented with 10% FBS, 4 mM L-glutamine, 1 mM sodium pyruvate, 100 U/mL penicillin–100 mg/mL streptomycin, and 400 ng/mL of hydrocortisone. Cells were maintained at 37 °C in a humidified incubator with 5% CO_2_ atmosphere.

### 2.6. Cytotoxic Assays

The cytotoxicity of the unloaded H_ALG_ and H_ALG-GO_ or the CUR-loaded CUR@H_ALG_ and CUR@H_ALG-GO_ was evaluated through microscopic observation and cell counting using the trypan blue method in a time-course experiment [48]. A small piece of hydrogel film of approximately 1.0 cm^2^ was placed inside a 35 mm tissue culture dish and hydrated overnight with the supplemented culture medium. The following day, the HBEpc and SCC-25 cells were trypsinized and counted, and 2 × 10^5^ cells were seeded (0 h). The cytotoxic effects were evaluated after 24 and 48 h of exposure with the hydrogel film.

## 3. Results and Discussion

### 3.1. Synthesis of the Hybrid Hydrogel and CUR Loading

Alginate hydrogels that were obtained via either chemical or physical crosslinking methods have been extensively explored as a platform for sustained release of drugs due to their capability of entrapping a wide range of bioactive molecules [43]. The achievement of physical crosslinking presents a significant advantage over chemical crosslinking strategies, since the latter often require the use of crosslinking agents, solvents, and other chemicals, which need to be removed at the end of the process [49,50]. 

In our experimental conditions, since a stable and homogenous dispersion of GO into the hybrid film was needed to ensure the formation of the hybrid GO system [51], a straightforward approach that did not involve the use of any toxic chemical species was adopted. In detail, a GO-to-ALG ratio of 2.0% (by weight) was chosen, since lower GO amounts could lead to the formation of less effective dosage forms, while higher ratios could result in an unstable dispersion in which large GO aggregates would be formed. This statement was confirmed by dynamic light scattering (DLS) measurements: the hydrodynamic diameter of GO@ALG (2%) in the reaction feed was found to be 287 nm, becoming 785 nm when a higher amount of GO (2.5%) was used. 

**Figure 1:**
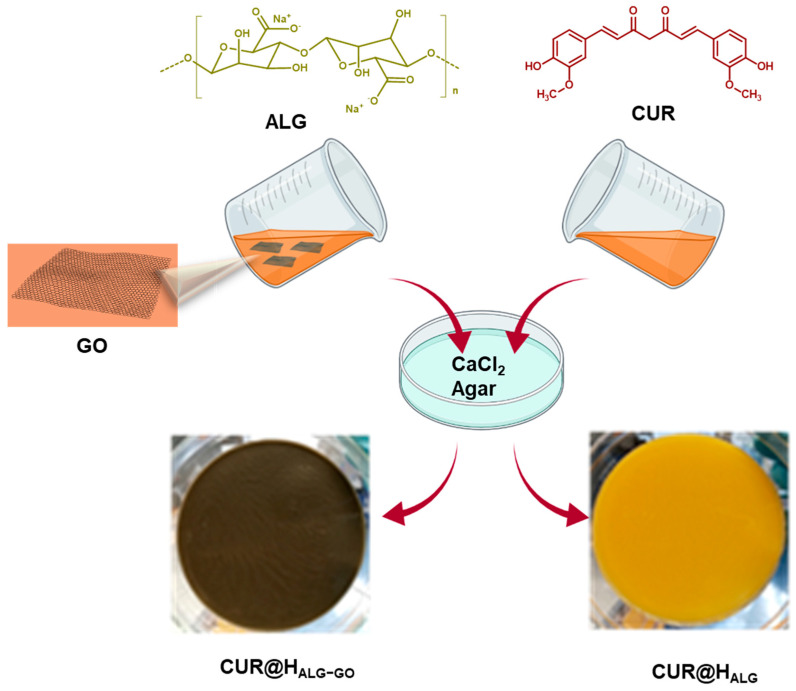
Schematic representation of CUR@H_ALG−GO_ synthesis.

Then, to promote the interaction of GO and CUR, a solution of CUR in ethanol (5 mg mL^−1^) was added while taking the need to use a lower amount of ethanol, which could otherwise cause alginate precipitation, into consideration. The crosslinking step was performed with the aid of an agar gel platform for calcium ion diffusion. With this method, the calcium ions slowly diffused into the polymeric blend [52], resulting in a homogenous and effective ionotropic gelation with the formation of CUR@H_ALG-GO_, as sketched in Figure 1. Control samples were prepared by using the same approach without the insertion of CUR (H_ALG-GO_ and H_ALG_) or GO (H_ALG_ and CUR@H_ALG_) into the reaction feed before the crosslinking step.

### 3.2. Characterization Procedure

Hybrid hydrogels were characterized with a multi-technique approach, allowing for the determination of different properties, including the morphological, chemical, and thermal properties, as well as the water affinity.

**Figure 2:**
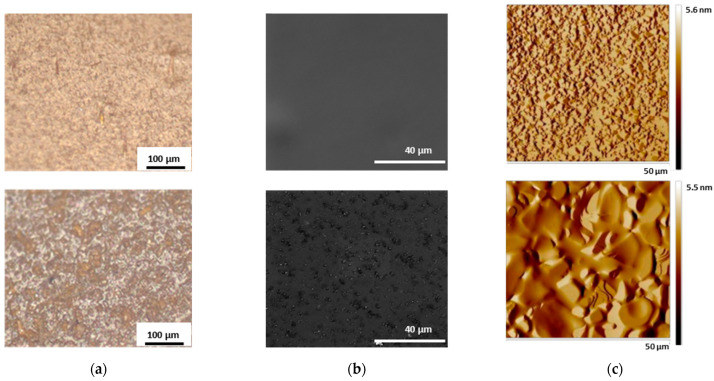
(**a**) Optical, (**b**) SEM, and (**c**) AFM images of CUR@H_ALG_ (top) and CUR@H_ALG-GO_ (bottom) showing: (**a**) the typical orange color of CUR loaded into the polymer networks; (**b**) the homogeneous incorporation of GO into the hybrid materials; (**c**) the presence of the GO sheet emerging from the hydrogel surface.

The investigation of CUR@H_ALG-GO_ with both optical and electron microscopy allowed for the effective incorporation of CUR and GO to be visualized (Figure 2). Moreover, homogeneous incorporation of GO into the polymer network was clearly evident by comparing the SEM images of the blank and hybrid systems (Figure 2b). The H_ALG_ sample showed a smooth surface, while the GO within H_ALG-GO_ appeared as embedded dots emerging from the hydrogel surface, as confirmed by the AFM images (Figure 2c).

**Figure 3:**
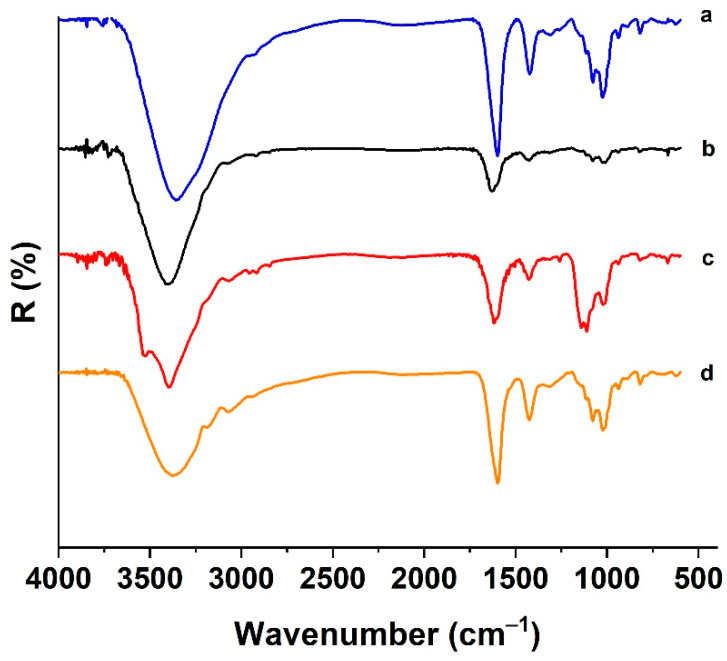
ATR-FTIR spectra of (**a**) ALG, (**b**) GO, (**c**) CUR, and (**d**) CUR@H_ALG-GO_.

The attenuated total reflectance Fourier transform infrared spectroscopy (ATR-FTIR) patterns of CUR@H_ALG-GO_ showed the presence of the typical absorption peaks of all components of the hybrid systems (Figure 3). 

The ALG showed bands at 3000–3600 cm^−1^ (stretching of OH bonds) and 1600–1422 cm^−1^ (asymmetric and symmetric stretching of carboxyl groups) [53]. The same groups were found in GO, with the stretching of OH and COOH located at 3399 and 1753 cm^−1^ [54], respectively, whilst the peak at 1074 cm^−1^ was attributed to the stretching of the C-O group. In the CUR spectrum, the signals at 3300–3550 cm^−1^ were assigned to the stretching vibrations of phenolic groups, with signals at 1466 and 1258 cm^−1^ corresponding to the stretching of aromatic C=C and bending of phenolic C-O, respectively [55].

**Figure 4:**
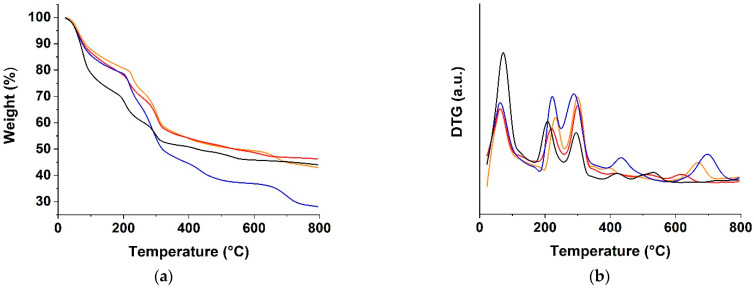
(**a**) TGA and (**b**) DTG curves of H_ALG_ (blue lines), H_ALG-GO_ (black lines), CUR@H_ALG_ (red lines), and CUR@H_ALG-GO_ (orange lines).

A thermogravimetric analysis (TGA) of the CUR, CA-GO, CA-CUR, and CA-GO-CUR hydrogels was performed in order to determine their thermal stability (Figure 4a). 

All of the curves showed a first weight loss at around 100 °C, which mainly correlated with the elimination of the free water. H_ALG-GO_ was characterized by the highest content of free water, with a weight loss of up to 30% in the 100–170 °C temperature range, a value that was reduced by 20% in the case of H_ALG_. The effect of the incorporation of GO into the polymer network was the result of the balance between the hydrophobic character of the GO sheets due to the sp^2^ carbon layer and the presence of oxygen-rich functionalities on the edge [56]. Here, the increased specific surface area of the hybrid hydrogels due to the incorporation of the polar surface of GO was responsible for the increased weight loss in the first temperature range. Further incorporation of highly hydrophobic CUR molecules into the CUR@H_ALG-GO_ sample was found to counter this effect. 

Moving to higher temperatures (200–300 °C), two degradation peaks related to the decomposition of free alginate chains could be observed, with the analysis of the derivative thermogravimetric (DTG) curve showing that the second peak was shifted to higher temperatures in the GO-containing samples (Figure 4b) due to the increased chain immobilization, resulting in a higher thermal stability. 

Above 300 °C, the fracture of glycosidic bonds and the decarbonylation of alginate chains led to the release of CO_2_ in addition to other small molecules and the consequent weight loss in the case of H_ALG_ [57]. These events were hindered in the other hydrogel samples as a consequence of the enhanced stabilization of the polymer structure by the presence of both CUR and GO. CUR and GO, indeed, were able to greatly affect the chain mobility and crosslinking density of the polymer networks.

**Figure 5:**
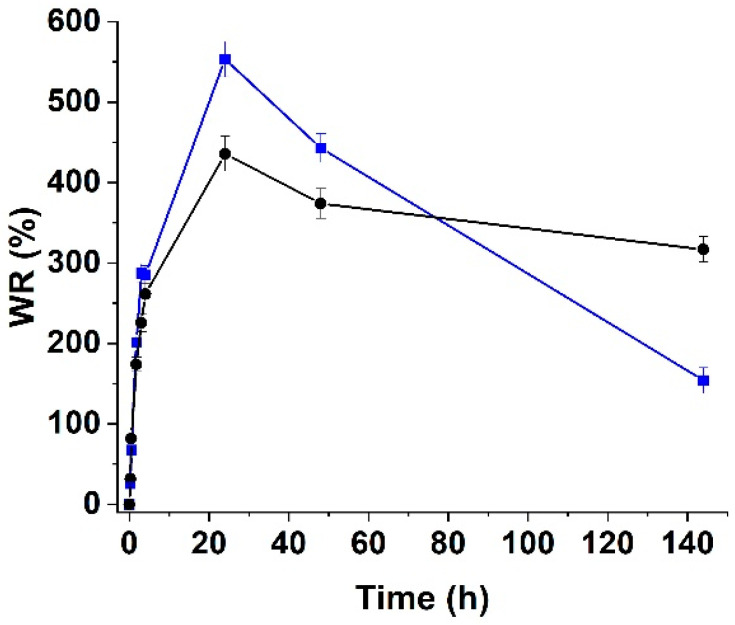
Swelling profiles of H_ALG_ (blue line) and H_ALG-GO_ (black line) at pH 7.4.

Then, swelling studies were performed in PBS at pH 7.4 to evaluate the water uptake and stability of the samples in a solution with an ion concentration comparable to that found in the human body (Figure 5). 

Due to the combination of two different effects, H_ALG-GO_ showed a reduced degree of swelling compared to H_ALG_. On one hand, it is well known that the swelling capacity of any kind of hydrogel sample decreases with an increase in the crosslinking degree, suggesting that the presence of GO inside the polymer network tightened the alginate chains, thus acting as a bridge. On the other hand, the hydrophobic effect of the C-C layer on the GO surface overcame the hydrophilicity conferred by the oxygen-rich groups when the exposure to water was longer than 2 h. 

Further extending the reaction time (>24 h) resulted in the destabilization of ALG networks due to the interference between calcium and sodium ions, thus resulting in the disruption of crosslinking and partial dissolution of alginate into the water solution with a reduction in the swelling degree [58]. Due to the incorporation of GO, the chain stabilization was found to counteract this event, thus resulting in a less pronounced reduction in the swelling properties.

### 3.3. Characterization of Biological Impact

**Figure 6:**
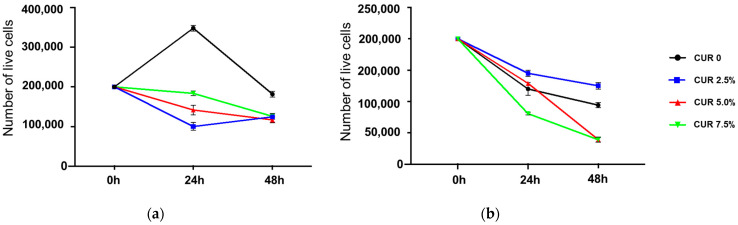
Cell viability profile of HBEpC 24 and 48 h after incubation with CUR-loaded (**a**) H_ALG_ and (**b**) H_ALG-GO_. Data are expressed as means ± SD of three counts.

To evaluate the efficiency of hybrid hydrogels as a delivery vehicle, three different CUR-to-hydrogel ratios (by weight) were used to incorporate the bioactive molecule, namely, 2.5, 5.0, and 7.5%. Then, the toxicity profile was investigated in normal human bronchial epithelial cells (HBEpC) in terms of cell viability after 24 and 48 h of incubation (Figure 6).

The analysis of the results clearly proved that the unloaded blank and hybrid hydrogels possessed completely different behaviors. H_ALG_ (Figure 6a) was found to be non-toxic and was well tolerated by HBEpC, with the number of living cells not being significantly reduced even after 48 h of incubation. Moreover, they could act to support cell growth and proliferation [59,60].

On the contrary, time-dependent toxicity was recorded in the case of H_ALG-GO_ (Figure 6b), with the viability (%) decreasing down to 50% after 48 h of incubation. These results may be related to both chemical and biological reasons. At first, the adopted synthetic strategy should be taken into consideration to explain the differences between the obtained results and the available data from the literature, which claim the complete non-toxicity of hybrid alginate- and graphene-oxide-based systems, including those of our research group [61]. In this study, the GO-to-hydrogel ratio (2%) was significantly higher than those previously employed (1.15%); thus, cells were exposed to different amounts of GO, and the crosslinking step consisted of a simple ionic gelation without the need for other reactants (such as the highly biocompatible acrylate monomers that were previously employed), which could also act as an obstacle to the direct interactions of the hydrogel and cell membrane.

**Figure 7:**
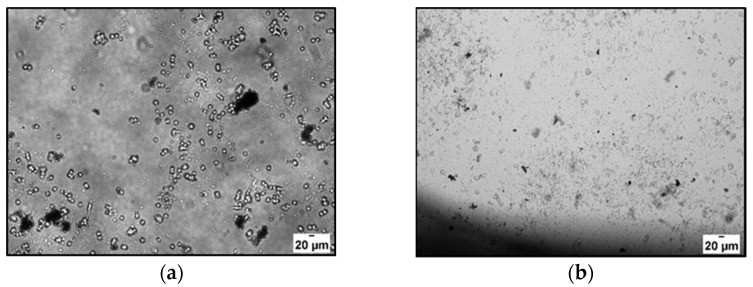
Optical microscope images of HBEpC after (**a**) 24 and (**b**) 48 h of incubation with H_ALG-GO_.

In our conditions, even after 24 h of incubation with H_ALG-GO_, the cells started to lose their shape, suggesting the induction of cell death, with the effect being more evident after longer incubation times (Figure 7).

When considering the biological effects arising from the hydrogel–cell interactions, it should be pointed out that although it is considered highly biocompatible, it has been reported that GO can show cytotoxic effects due to its interference with the cell redox state, mainly in the extracellular media [62]. This is only partly related to the structure of GO, which consists of a π–π surface layer with oxygen-rich functionalities and can exert either pro- or antioxidant properties depending on the environmental conditions [63]. The main determining phenomenon at the basis of GO’s cytotoxicity is due to its adsorption properties: GO can indeed adsorb nutrients from the extracellular compartment, thus inducing nutrient depletion and oxidative stress at relatively high GO concentrations [64]. Moreover, it was reported that GO is able to induce the production of reactive oxygen species (ROS) in cell culture media, which is a further confirmation of the extracellular nature of GO’s toxicity [65].

In our condition, the oxidative nature of cell toxicity was confirmed when considering the effect of CUR-loaded samples, with the effect on cell viability being a function of both CUR concentration and incubation time. CUR@H_ALG-GO_ 2.5% (Figure 6b, blue lines) was found to be less toxic than H_ALG-GO_ after 24 and 48 h of incubation. When considering CUR@H_ALG-GO_ 5.0% (Figure 6b, red lines), the protective effect was recorded only after 24 h, while an increase in toxicity was recorded with longer incubation times. The antioxidant/prooxidant paradox can help understand this phenomenon, since it is well known that higher concentrations of antioxidant species in an active redox environment can lead to an increase in oxidative stress, rather than a decrease [66,67]. The same hypothesis can be used to explain the increased cell toxicity of CUR-loaded H_ALG_ samples at all tested CUR concentrations.

**Figure 8:**
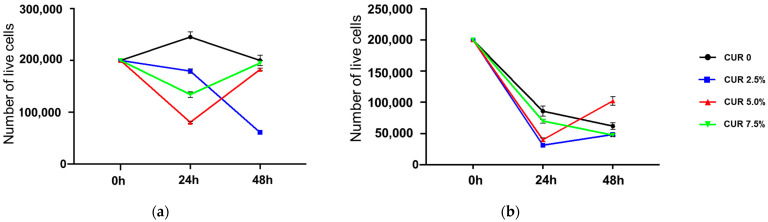
Cell viability profile of SCC-25 cells after 24 and 48 h of incubation with CUR-loaded (**a**) CUR@H_ALG_ and (**b**) CUR@H_ALG-GO_. Data are expressed as means ± SD of three counts.

Next, we investigated the viability of cancerous SCC-25 cells upon incubation with unloaded and loaded hydrogels (Figure 8).

**Figure 9:**
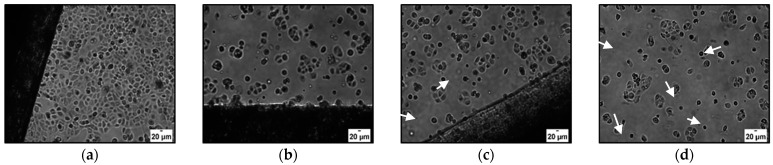
Microscope images of SCC-25 cells 24 h after incubation with (**a**) H_ALG-GO_, (**b**) CUR@H_ALG-GO_ 2.5%, (**c**) CUR@H_ALG-GO_ 5.0%, and (**d**) CUR@H_ALG-GO_ 7.5%. Arrows indicate apoptotic cells.

The effects of unloaded the H_ALG_ and H_ALG-GO_ samples (Figure 8, black lines) were similar to those recorded for the HBEpC, with a higher toxicity recorded for H_ALG-GO_. More interesting results were obtained when the CUR-loaded samples (2.5%) were used. Here, the polyphenol was found to counteract cell death upon loading on H_ALG_, while the cytotoxicity was retained and even enhanced when H_ALG-GO_ was used as a delivery device (Figure 9). It is evident, indeed, that either the cell number or morphology was altered with increased CUR concentrations, suggesting the induction of cell death. 

To explain this behavior, it should be considered that, like many other polyphenols, CUR was proved to be highly toxic for cancer cells while being tolerated by healthy cells due to the different cell redox states [68]. Thus, the final effects of CUR-loaded hydrogels were the result of the alteration of the cells’ redox state at either the extracellular or intracellular level, resulting in pronounced anticancer activity and reduced toxicity in healthy cells. 

These results are very promising for future applications, although further experiments are required to properly understand the biological pathways involved and to design more effective systems to be applied in in vivo experiments.

### 3.4. CUR Release Experiments

To better understand the results of the biological impact of the synthesized hydrogels and to evaluate the possibility of guaranteeing a controlled CUR release over time, we extensively investigated the CUR release profiles of the H_ALG_ and H_ALG-GO_ samples. Samples loaded with the highest ratios of CUR (5.0 and 7.5%) were found to possess a significantly high burst release in the first experimental times, reaching *M_t_*/*M*_0_ values of 0.9 (H_ALG_) and 0.7 (H_ALG-GO_) after 2.0 h; thus, they were not investigated in this characterization.

**Figure 10:**
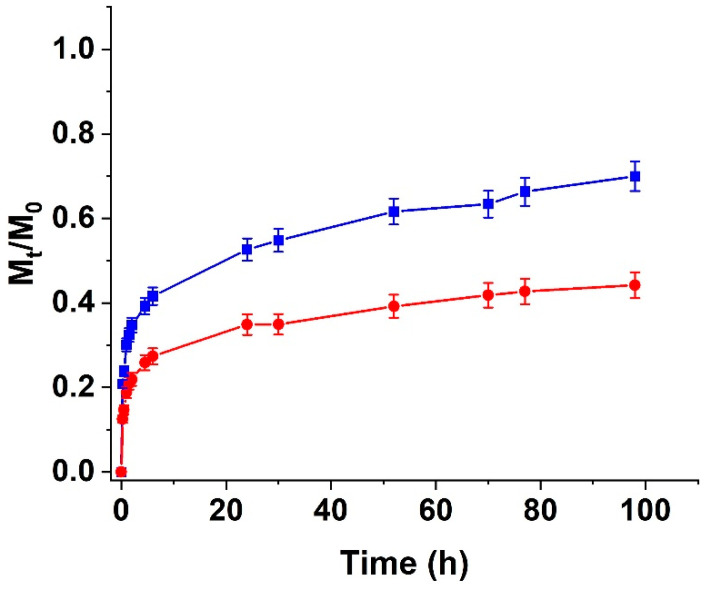
CUR release from H_ALG_ (blue line) and H_ALG-GO_ (red line) as a function of time. The CUR-to-hydrogel ratio (by weight) was 2.5%.

The release profiles of the 2.5% CUR-loaded samples are depicted in Figure 10.

As expected, the highest affinity of GO for CUR, resulting in π-π staking interactions between the aromatic rings of the polyphenols and the sp^2^ carbon layer of the carbon nanostructure, led to a more sustained release in the case of the hybrid H_ALG-GO_ sample, with *M_t_*/*M_0_* values that did not reach 50% even after 96 h (>70% in the case of H_ALG_). 

Suitable mathematical models for the determination of the release kinetics helped in understanding the mechanisms of drug release.

In detail, we applied four different models, namely, the zero-order, first-order, Ritger–Peppas, and Peppas–Sahlin equations [69,70]. 

For the analysis of the kinetic parameters, we applied nonlinear methods to avoid any distortions created in the error distribution by a logarithmic linearization approach.

The first equation applied was the zero-order kinetic model (Equation (3)):

(3) $$\frac{M_{t}}{M_{0}}=k_{0}t$$

Then, the first-order kinetic model was applied (Equation (4)):

(4) $$\frac{M_{t}}{M_{0}}=a\left(1-e^{-k_{1}t}\right)$$

Model 3 was described by the Ritger–Peppas Equation (5):

(5) $$\frac{M_{t}}{M_{0}}=k_{R}t^{n}$$

Model 4 was given by the Peppas–Sahlin Equation (6):

(6) $$\frac{M_{t}}{M_{0}}=k_{S}^{\prime }t^{m}+k_{S}^{\prime \prime }t^{2m}$$

Here, *t* is the release time; $k_{0},$ $k_{1}$*,*$k_{R}$*,* $k_{S}^{\prime }$*,* and $k_{S}^{\prime \prime }$ are the zero-order, first-order, Ritger–Peppas, Fickian diffusion, and anomalous diffusion kinetic constants; *a* is the first-order release coefficient; *n* is the Ritger–Peppas coefficient.

In zero-order kinetics that indicate a steady-state concentration profile, the release kinetics are independent of the concentration of the released drug; variation in concentration did not affect the observed drug diffusion, with the release being very slow. On the contrary, first-order kinetics indicated concentration-dependent processes in which the difference between the drug concentration inside and outside the dosage form acted as the driving force [69]. For swelling-controlled delivery vehicles, the semiempirical “power law” model proposed by Ritger and Peppas can be applied [70]. This model assumed an exponential proportionality between the amount of drug released and time, with the exponent describing the release mechanism driven by a Fickian diffusion (*n* ≤ 0.50 for hydrogel films) or anomalous transport processes (0.50 *n* < 1.0). Finally, the model proposed by Peppas and Sahlin allowed the contributions of Fickian and anomalous phenomena to be quantified over the release rate depending on the prevalence of $k_{S}^{\prime }$ (Fickian diffusion) or $k_{S}^{\prime \prime }$ (anomalous contributions) [70].

In our conditions, for both H_ALG_ and H_ALG-GO_, it was clearly evident that the release mechanism was governed by the diffusion of CUR molecules through the swollen hydrogel matrix, since for both zero- and first-order kinetics, R^2^ values lower that 0.85 were recorded (Table 1). 

On the contrary, a good fit was obtained between the experimental and theoretical data when Equations (4) and (5) were applied (R^2^ > 0.99 in all cases). Finally, the determination of the $k_{S}^{\prime }/k_{S}^{\prime \prime }$ ratio allowed for the effect of the incorporation of the GO nanosheet within the polymer network to be highlighted. For both the H_ALG_ and H_ALG-GO_ samples, indeed, the prevalence of $k_{S}^{\prime }$ over $k_{S}^{\prime \prime }$ was recorded, but the hybrid hydrogel showed a more significant involvement in anomalous phenomena, since the $k_{S}^{\prime }/k_{S}^{\prime \prime }$ ratio was reduced to almost half when compared to samples that not did not contain the carbon nanostructure. This can be attributed to the strong affinity of CUR for GO, which modified the release mechanism due to the involvement of π–π staking interactions between drug and delivery vehicles, while weak hydrogen bonds were mainly involved in the loading of CUR into H_ALG_.

## 4. Conclusions

In this study, we presented experimental evidence that a novel hybrid H_ALG-GO_ hydrogel can be used as a valuable platform for controlling the release of bioactive agents, such as CUR, due to the higher affinity of the GO surface for aromatic species. 

As a synthetic strategy, an ionic crosslinking by means of calcium ions was selected to increase the amount of GO being incorporated within the polymer network and to avoid the use of any other chemical species. Suitable physico-chemical characterization allowed the assessment of the homogeneous incorporation of all hydrogel components and the determination of their influence on the morphological, thermal, and water-affinity behaviors of the hybrid system. In detail, GO was found to increase the thermal stability and to reduce the loss of integrity in water media, while CUR was able to enhance the biocompatibility whilst simultaneously acting as a powerful anticancer agent in SCC-25 cancer cells, with the cytotoxic efficiency being strictly related to the CUR-to-hydrogel ratios. The determination of the CUR release profiles, which were extensively investigated through suitable modeling, clearly proved that the presence of GO led to a prolonged CUR release, suggesting the possibility of extending the therapeutic efficiency of the delivery device over time. 

Further experiments will be performed to assess the ex vivo and in vivo suitability of the hybrid hydrogels, as well as to determine the most effective CUR loading amount.

## Figures and Tables

**Table 1:** Kinetic parameters of CUR release from hybrid and blank ALG hydrogels.

**Sample**	**Zero Order** **(Equation (3))**		**First Order** **(Equation (4))**		Ritger–Peppas (Equation (5))			Peppas–Sahlin (Equation (6))				
	**R^2^**	***k*_1_** (**10^−3^**)	**R^2^**	***k_2_***	**R^2^**	***k_R_***	***n***	**R^2^**	***m***	$k_{S}^{\prime }$	$k_{S}^{\prime \prime }$ (**10^−2^**)	$\frac{k_{S}^{\prime }}{k_{S}^{\prime \prime }}$
H_ALG_	0.692	9.36	0.794	0.52	0.996	0.29	0.19	0.998	0.21	0.31	1.7	18
H_ALG-GO_	0.690	6.02	0.826	0.38	0.993	0.19	0.19	0.997	0.24	0.20	2.0	10

## Data Availability

Not applicable.
